# Identification of sunflower, rapeseed, flaxseed and sesame seed oil metabolomic markers as a potential tool for oil authentication and detecting adulterations

**DOI:** 10.1371/journal.pone.0284599

**Published:** 2023-04-20

**Authors:** Agata Sumara, Anna Stachniuk, Marta Olech, Renata Nowak, Magdalena Montowska, Emilia Fornal

**Affiliations:** 1 Department of Bioanalytics, Medical University of Lublin, Lublin, Poland; 2 Department of Pharmaceutical Botany, Medical University of Lublin, Lublin, Poland; 3 Department of Meat Technology, Poznan University of Life Sciences, Poznan, Poland; Bangabandhu Sheikh Mujibur Rahman Agricultural University, BANGLADESH

## Abstract

Testing the composition, quality and authenticity of edible oils is crucial to safeguard the consumers’ rights and health. The aim of our study was to identify oil-specific markers to enable the differentiation and authentication of sunflower, sesame, flaxseed and rapeseed oils, and to evaluate their antioxidant activity, total phenolic and carotenoid content. A metabolomic approach based on liquid chromatography coupled to quadrupole-time-of-flight mass spectrometry was employed for marker discovery. Spectrophotometric method was used for determination of antioxidant activity, total phenolic and carotenoid content. 76 oil samples from the four different manufacturers were examined. We identified 13 oil-specific markers for sunflower seed oil, 8 for rapeseed oil, 5 for sesame seed oil and 3 for flaxseed oil, their retention times, accurate masses, and characteristic fragment ions are reported. The abundances of the markers for each plant species were found to vary depending on the oil producer and the product batch. Significant differences in antioxidant activity, total phenolic and carotenoid content were also observed both between oils and within oil type. The highest total phenolic content (84.03 ± 4.19 to 103.79 ± 3.67 mg of gallic acid/kg) and antioxidant activity (245.67 ± 7.59 to 297.22 ± 2.32 mg Trolox/kg) were found in sesame seed and flaxseed oils, respectively. Identified metabolic markers can be used as qualitative markers to confirm the authenticity or to detect adulterations of oils. Composition, properties and authenticity testing should be more rigorous for food products marketed as health-promoting.

## Introduction

The production of edible plant seed oil is increasing globally. In antiquity, many plants and plant oils were used as medicines, paints, perfumes, cosmetics and food. Today, products from oil plants are used as raw materials in biofuel production in the petrochemical industry [1]. However, plant oils are used primarily in the food industry, as more than 75% of lipids in the human diet come from edible vegetable oils [2]. The flavour, smell and health value of these products stem from their significant content of vitamins, minerals, fatty acids, phenols, sterols, tocopherols and carotenoids [3]. For this reason, vegetable oils are used in pharmaceutics and medicines and have a potentially beneficial effect in the treatment of many illnesses, such as liver disease [4], knee osteoarthritis [5] and hypertension [6].

The increase in global oilseed production doubled between 1975 and 2019. According to 2014–2018 statistical data, rapeseed (12%) and sunflower (9%) were second and third in terms of global vegetable oil production, preceded by soybean (60%) [7]. Rapeseed oil is used in the food and animal feed industries, and in biodiesel production. The wide availability of rapeseed oil in Europe means that rapeseed oil comprises 42% of the total consumed plant oils in the European Union and is the most commonly used oil in the food industry [8]. Sunflower seed oil is produced on a large scale, particularly in Ukraine, Russia and Argentina, and almost two thirds of global production is concentrated in Europe [7]. In Poland, sesame and flaxseed oils are more expensive than sunflower or rapeseed oils, so confirmation of product authenticity has a huge financial impact on the food industry. More expensive oils can be surreptitiously replaced by cheaper products, or oils with a lower commercial value can be added to high-quality oil products [9]. Because of their widespread use and economic importance, edible plant seed oils should be verified to ensure their high quality. As reported by Issaoui and Delgado plant oils are strategic products that forces governments to take regulatory measures with respect to controlling the marketing of edible oils [10]. The health aspects are another, even more important factor, reinforcing the importance of authentication, as recent studies show a significant increase in allergies identified after the consumption of products containing sesame [11].

Edible plants naturally produce a variety of compounds of different chemical nature that are used for plant growth and development [12, 13]. Low molecular weight phytochemicals are classified into three groups: primary metabolites, secondary metabolites and hormones [14]. Proper plant development depends on their presence and concentration. Metabolomics enabled the systematic study of metabolites and their interactions [15]. Metabolomics studies were successfully applied in pharmaceutical, biomedical, agricultural and nutritional sciences, including drug discovery, disease diagnosis and plant phenomena [16]. More recently, the metabolomics approach were found to be an essential technique for food authentication [13, 15, 17]. One of main areas of the application of metabolomics in the food industry focuses on the discovery of food identity markers. Metabolomic markers were reported, among others, for detecting adulteration of edible oils. Filbertone and 4, 40 -dimethylsterols were used as markers for the detection of hazelnut oil in virgin olive oils [18, 19]. Delta 7-stigmastenol was used as a marker for the detection of sunflower or soybean oil in olive oil [20]. 11 metabolites related to the biosynthesis of isoflavonoids were detected in soya bean oils but not in rapeseed oils [13].

Researchers developed several instrumental analytical methods to date to identify the adulteration of and authenticate edible plant oils. Methods used to detect the adulteration of sesame seed oil include nuclear magnetic resonance spectroscopy [21], ion mobility spectrometry [22] and fluorescence spectroscopy [23]. Flaxseed oil was analysed using gas chromatography mass spectrometry, GC/MS [24] or mid-infrared spectroscopy together with a chemometric technique [25]. The triacylglycerol fingerprint of edible oils were analysed using liquid chromatography mass spectrometry, LC/MS [26]. A wide variety of analytical methods enable the selection and easier and faster implementation of methods to routine oil testing by delivering solutions which meet the needs and technical equipment capabilities of a laboratory. Thus, further research efforts are required to develop new analytical tools and techniques that are fast, efficient and reliable to detect adulteration and authenticate edible vegetable oils.

The aim of the study was the identification of metabolomic markers specific to oils cold-pressed from seeds of sunflower (*Helianthus annuus* L.), rapeseed (*Brassica napus* L.), sesame (*Sesamum indicum* L.) and flax (*Linum usitatissimum* L.), which will be useful for oil authentication and the detection of adulterations. The metabolomic analyses were carried out using liquid chromatography high resolution mass spectrometry; fingerprints were acquired a on a quadrupole time of flight mass spectrometer (LC/Q-TOF MS). Oil samples of the four oil plant species sourced from four oil producers were investigated. Additionally the antioxidant activity of oils, total phenolic and carotenoid contents was evaluated using spectrophotometric methods for the oil bioactivity characterisation.

## Materials and methods

### Materials

Formic acid (LC–MS grade) was obtained from Merck KGaA (Darmstadt, Germany). Acetonitrile and methanol (both Optima® LC-MS grade) were purchased from Thermo Fisher Scientific (Waltham, MA, USA). Extracts were filtered through a 0.20 μm, 4 mm titan syringe filter (Thermo Fisher Scientific). Ultra-pure water was obtained from a water purification system (Millipore Direct-Q3-UV, Merck KGaA, Darmstadt, Germany). Ethanol, hexane, glacial acetic acid and sodium carbonate anhydrous were obtained from Avantor Performance Materials Poland (Gliwice, Poland). Gallic, oleanolic acid, *β*-carotene, vanillin, 2,2′-azino-bis-3(ethylbenzthiazoline-6-sulphonic acid) (ABTS) and Trolox were purchased from Sigma-Aldrich Chemical Co. (St. Louis, Mo, USA). Perchloric acid and Folin-Ciocalteu reagent were obtained from Chempur (Piekary Śląskie, Poland).

### Samples

We used 32 cold-pressed seed oils—flax (*Linum* L.), sesame (*Sesamum* L.), sunflower (*Helianthus* L.) and rapeseed (*Brassica napus* L.)—acquired from four oil manufacturers in Poland (a, b, c, and d). Two bottles of each of the four kinds of oil were purchased from each of the four manufacturers and the bottles had different lot numbers. To verify the specificity of metabolic markers, two bottles of pumpkin seed oil, evening primrose oil, black cumin oil, hemp seed oil and thistle oil—a total of 40 products of unrefined, cold-pressed vegetable oils—were purchased from the same manufacturers for analysis. All oils were stored in a refrigerator (4°C) in their original bottles until analysis.

### Preparation of the oil mixtures

Two-component mixed oil samples were prepared at 1% [v/v] and 5% [v/v]. From each purchased bottle of flaxseed, sesame or rapeseed oil, 5 μl of the oil were pipetted for the 1% mixes and 25 μl for the 5% mixes, and then 495 and 475 μl of sunflower oil were added, respectively. The sunflower oil mixes were prepared in the same way as the other mixes, in this case the rapeseed oil was used as a diluent. The mixtures were vortexed for 1 min.

### Extraction

Samples for LC/MS metabolomic analyses by were prepared by liquid-liquid extraction according to a method developed by Becerra-Herrera et al. [27], with several modifications. Samples of each oil (0.5 ml) and 0.5 ml aliquots of oil mixtures were mixed with 0.5 ml methanol-water (80:20, v/v) in 2-ml Eppendorf tubes. The samples were shaken by hand for 30 s, vortexed for 2 min and then centrifuged for 5 min at 12100 g. Next, 0.3 ml of the upper phase was transferred to a new 2-ml Eppendorf tube, mixed with 0.45 ml ultrapure water and shaken by hand for 30 s. The samples were stored for 15 min at 4°C, then centrifuged for 30 min at 12100 g. The obtained liquid was filtered through a titan syringe filter (0.2 μm). The extracts were subjected to LC/MS analyses and to total phenol content analysis by the Folin-Ciocalteu colorimetric method.

### LC/Q-TOF MS analysis

Metabolomic analysis was performed on a high-performance liquid chromatograph (1290 Infinity, Agilent Technologies, Santa Clara, CA, USA) coupled with a 6550 iFunnel Q-TOF mass spectrometer (Agilent Technologies) equipped with an ion source (Jet Stream Technology, Agilent Technologies) operated in positive mode (ESI+). The metabolites were separated using a RRHT Zorbax Extend C18 column (2.1 × 100 mm, 1.8 μm, Agilent Technologies). The LC parameters were an injection volume of 5 μl and a column temperature of 45°C. The mobile phase consisted of 0.1% formic acid in water (A) and 0.1% formic acid in acetonitrile (B) with a flow rate of 0.4 ml/min. Elution was performed for 30 min with a 2 min post run at 3%B. The gradient elution conditions were 0–25 min, 3%B to 95%B; 25–30 min, 95%B. The ion source (nitrogen) temperature was 225°C; flow rate: 12 l/min; nebulizer pressure: 50 psi; sheath gas temperature: 275°C; sheath gas flow: 12 l/min; and capillary voltage: 3500 V. The nozzle voltage was set at 1000 V and the fragmentor voltage at 275 V; the mass scan mode range was *m/z* 100–1700. Internal mass calibration was enabled by using two reference masses at *m*/*z* 121.0509 and *m*/*z* 922.0098. MS scans and targeted MS/MS scans were acquired; collision energies were set in the range of 10-50V. The LC-QTOF was controlled by Agilent MassHunter Data Acquisition software (B.09.00), and data were processed with Agilent Mass Hunter Qualitative analysis software (B.10.00).

### LC/Q-TOF MS data processing and multivariate analysis

Bioinformatic analysis to identify specific metabolomic markers for plant oils was carried out on raw LC/MS data processed using Molecular Feature Extractor in Agilent MassHunter Qualitative Analysis Software with limits of the used peak height set to 600 counts, compound absolute count set to 50,000 counts and quality score ≥ 80. The obtained datasets of compounds (metabolic features) containing the mass-to-charge ratio (*m/z*), retention time (RT) and peak height were converted to the CEF file format and imported to Mass Profiler Professional (MPP) software. All features were first aligned using a 0.1% + 0.15 min RT window, and 5 ppm + 2.0 mDa mass window. In the second step, the aligned samples were analysed using the Filter by Flags and Filter by Frequency algorithms and the lists of differentiating metabolic features were obtained. The files were imported into a multivariate data analysis program (SIMCA-P version 16, Sartorius, Goettingen, Germany). Two types of mutivariate analyses were employed: principal component analysis (PCA-X, unsupervised) and orthogonal partial least-squares discriminate analysis (OPLS-DA). Data preprocessing involved Pareto scaling and centering. Models were cross-validated (PCA, OPLS-DA) and validated by permutation testing (OPLS-DA). The quality of models was assessed basing on model statistics: R2X, R2Y, S2Y, Q2 and SEE from cross-validation, Model performance was evaluated by considering the explained variation R2—goodness of fit for X- and Y-variables, respectively, the predictive variation Q2—goodness of prediction, the fit for predicted variables, the variance of Y-matrix (S2Y), i.e., the residual (not modelled) variance of Y and the standard error of estimate (S.E.E.)—a root-mean-square error of estimates.

### Analysis of antioxidant activity using the ABTS^•+^ radical

Antioxidant activity was determined according to a modification of the method of Olech et al. [28]. In brief, 20 μl of oil diluted with hexane (3- to 100-fold dilutions) was mixed with 180 μl of ABTS^•+^ s,olution (0.096 mg/ml in methanol: ethyl acetate; 1:1 v/v). The mixtures were shaken and incubated at 28°C for 6 min, then the absorbance was measured at 734 nm using a microplate reader (Infinite Pro 200F, Tecan Group Ltd., Männedorf, Switzerland). The radical scavenging activity was calculated using the following formula:

%Reduction=[(Ac−As)/Ac]×100

where Ac is the absorption of the control sample (ABTS^•+^ solution and solvent instead of the sample), and As is the absorption of the sample with the ABTS^•+^ reagent.

The EC_50_ value (sample concentration required to decrease the absorbance of ABTS by 50%) was determined for every sample based on a dose-response curve. The results were expressed as standard equivalents using Trolox (TEAC; Trolox equivalent antioxidant capacity) based on its EC_50_ value obtained under the same conditions.

### Total phenolic content

Total phenolic content was determined according to a slight modification of the method of Olech et al. [28]. In brief, 20 μl of oil methanolic extract, a blank (methanol) and gallic acid standard solutions were mixed with 20 μl of Folin-Ciocalteu reagent and 160 μl of sodium carbonate solution (75 g/l) on 96-well transparent microplates (Nunclon, Nunc; Roskilde, Denmark), then absorbance was measured after 20 min at 680 nm using the microplate reader. The results were compared with a standard curve prepared under the same conditions for a series of dilutions (25 to 200 μg/ml) of gallic acid solution and expressed as mg of gallic acid per kg of oil. Triplicate measurements were made for each sample and averaged.

### Total carotenoid content

Oils and *β*-carotene (standard) were diluted in ethanol: hexane (1:1; v/v) before analysis. The absorbance was measured at 460 nm in an Evolution 300 spectrophotometer (Thermo Scientific, Lafayette, LA, USA) using hexane: ethanol (1:1; v/v) as a blank. The results were expressed as mg of *β*-carotene per kg of oil using the calibration curve prepared for *β*-carotene at concentrations ranging from 5 to 500 μg/ml. The analyses were performed in triplicate.

### Statistical analysis

Statistical analysis was performed using Statistica 14.0 (StatSoft, TIBCO Software, Pao Alto, CA, USA). One-way ANOVA and Tukey HSD test were employed, the significance level was set at p > 0.05.

## Results and discussion

### Identification of characteristic markers

Flaxseed, rapeseed, sunflower and sesame seed oil metabolomic features were extracted using the find by molecular feature algorithms, aligned and profiled. For each oil, only compounds found in all of the analysed samples were used in subsequent steps. A list of compounds, annotated by the accurate *m/z* and the retention times was exported to a CSV file for further manual examination and into CEF files for multivariate data analysis. To ensure that each metabolomic feature derives from the selected plant species, we compared the ions with blank samples containing only organic solvents used in sample preparation, and processed the same way as oils, to exclude ions not originating from oil plants.

**Fig 1:**
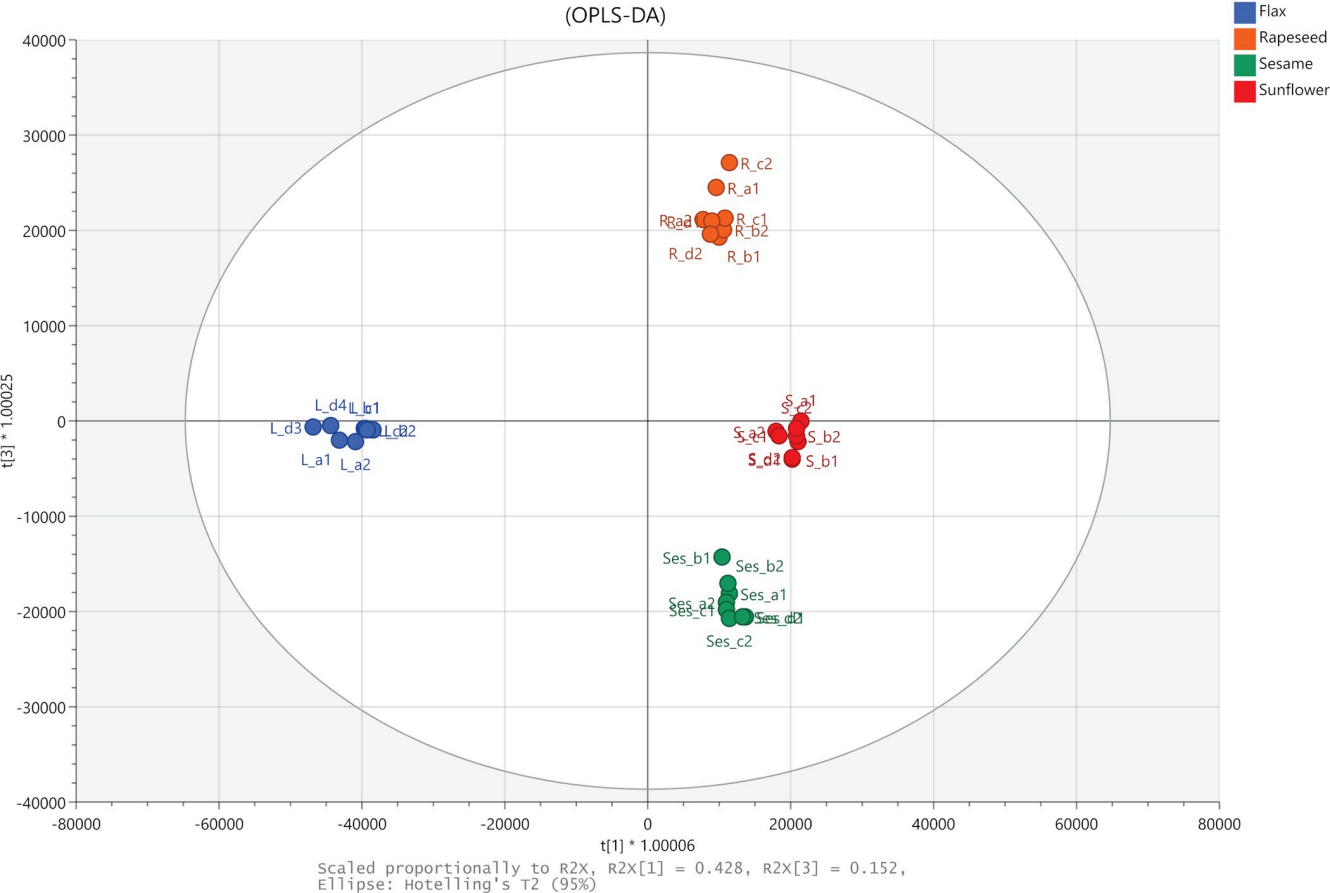
Differentiation of four seed oils based on metabolomics profiles. OPL-DA t[1]-t[3] score plot. Model R2X = 0.878, R2Y = 0.99, and Q2 = 0.981; R2X[1] = 0.428, R2X[2] = 0.223, R2X[3] = 0.152.

To evaluate the potential of metabolic profiles to classify and discriminate oils multivariate data analyses were performed. First the PCA was applied to examine if the systematic variation in metabolic profiles between oils exists. It appeared that samples of the same type of oil grouped together in different parts of PCA score plots. Clear separation of oils was observed indicating high potential of classification and discriminating power of oil metabolomic profiles. Next, a OPLS-DA model was build; OPLS separates the systematic variation in dataset into two parts, one part that is correlated (predictive) to Y (oil type) and one part that is uncorrelated (orthogonal) to Y. OPLS*-DA* modeling resulted in the model with three predictive components (3+2+0). Model R^2^X, R^2^Y and Q^2^ were, respectively, equal to 0.878, 0.99 and 0.981. Fig 1 shows the t[1]-t[3] score plot of the model.

Subsequent examination of OPLS-DA contribution plots allowed to select metabolic markers characteristic to sunflower seed oil, rapeseed oil, sesame seed oil and flaxseed oil. To verify their specificity, metabolic features of each oil were compared to eight other oil species (the list of oils is included in the section Samples; in total 64 samples for the negative test for each oil were analysed) to exclude common ions. The marker (characteristic to the oil) ions were then fragmented using various collision energies (10-50V).

**Fig 2:**
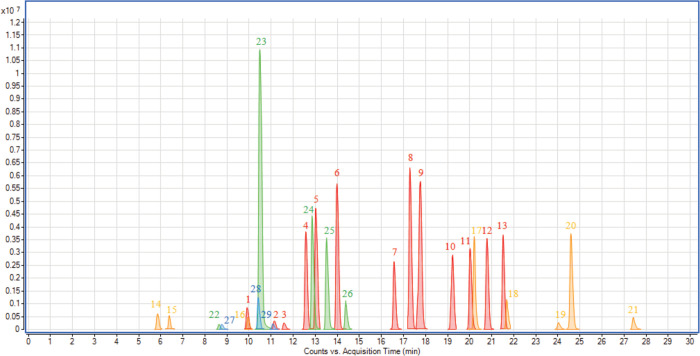
LC–QTOF extracted ion chromatogram of 29 metabolomic oils markers. Sunflower–red, rapeseed–yellow, sesame–green, flax–blue. The number of analytes corresponds to those given in Table 1.

**Fig 3:**
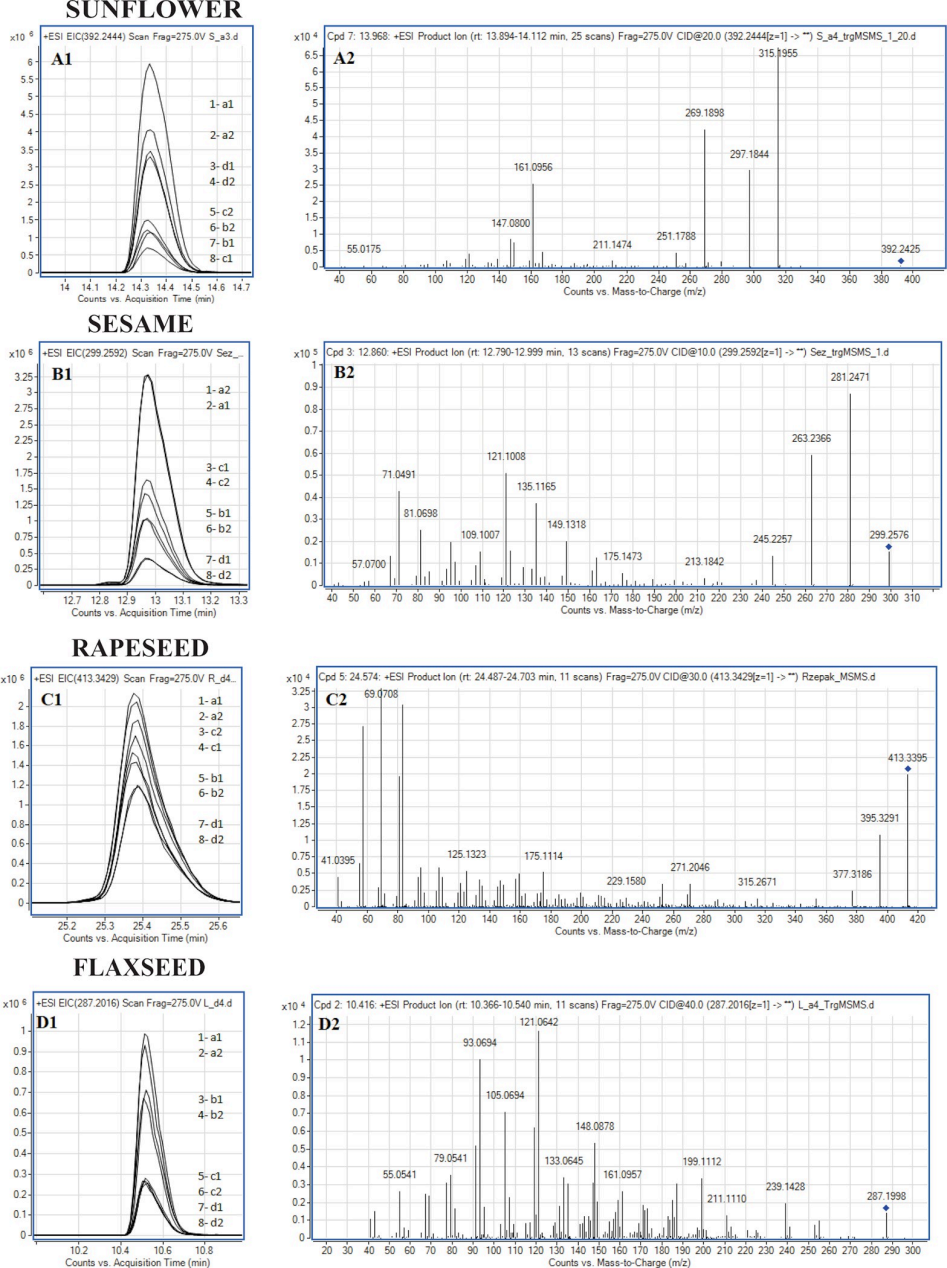
Extracted ion chromatograms (EIC) and MS/MS mass spectra of metabolomic markers for sunflower seed oil, sesame oil, rapeseed oil and flaxseed oil. EIC for eight different oil samples (manufacturers marked by a-d letter, batch by 1–2 number) are overlaid.

We discovered 13 metabolomic markers characteristic for sunflower seed oil, 8 for rapeseed oil, 5 for sesame seed oil and 3 for flaxseed oil, they are shown on the chromatogram in Fig 2. All markers were searched against databases: the Food Component Database (www.foodb.ca), the Human Metabolome Database (www.hmbd.ca), and PhytoHub (www.phytohub.eu), MassBank of North America (https://mona.fiehnlab.ucdavis.edu), METLIN Gen2 (Mass Consortium Corporation) MassBank Europe (https://massbank.eu/MassBank), MassBank Japan (http://www.massbank.jp) yet the search did not yield the identification result. Plant metabolic richness coming among others from plant genes, multiple substrate specifications for enzymes, subcellular compartmentation, and the occurrence of nonenzymic reactions is the cause of databases to be still far from completed. Oil-specific metabolic markers were characterised to fragmentation spectrum level; retention times, accurate masses of precursor ion and fragment ions are reported for each marker in Table 1. The example fragmentation spectra are presented in Fig 3.

**Table 1:** Metabolomic oil markers detected by LC-QTOF-MS/MS.

	No.	*m/z* [M+H]+	RT [min]	MS/MS [*m/z*]	CE [V]	1%	5%
**SUNFLOWER OIL**	**1**	231.1386	10.03 ± 0.18	185.1318, 143.0847, 131.0851, 159.1166, 173.0958	20	+	+
	**2**	217.0865	11.31 ± 0.25	43.0179, 189.0539, 163.0382, 91.0540, 147.0793	20	+	+
	**3**	345.0975	11.77 ± 0.25	315.0495, 182.9918, 133.0644, 287.0544, 99.0073	40	+	+
	**4**	203.1076	12.76 ± 0.24	43.0181, 77.0385, 91.0539, 145.0642, 107.0487	40	+	+
	**5**	301.2172	13.15 ± 0.2	255.2104, 119.0851, 107.0853, 145.1007, 133.1008	20	+	+
	**6**	392.2444	14.14 ± 0.26	315.1955, 269.1898, 297.1844, 161.0956, 147.0800	20	+	+
	**7**	420.2758	16.76 ± 0.3	315.1955, 269.1898, 297.1845, 161.0956, 251.1790	20	+	+
	**8**	432.2758	17.47 ± 0.28	315.1957, 269.1899, 297.1845, 161.0956, 397.2364	20	+	+
	**9**	434.2913	17.98 ± 0.33	315.1957, 297.1846, 296.1899, 161.0957, 333.2044	20	+	+
	**10**	576.3394	19.44 ± 0.26	541.3012, 457.2439, 499.2907, 57.0697, 85.0645	10	+	+
	**11**	590.3552	20.23 ± 0.31	555.3190, 471.2605, 57.0696, 369.1917, 85.0645	20	+	+
	**12**	604.3699	20.97 ± 0.3	485.2759, 569.3337, 57.0697, 85.0646, 383.2070	20	+	+
	**13**	618.3865	21.74 ± 0.26	499.2917, 57.0696, 583.3493, 85.0644, 397.2225	20	+	+
**RAPESEED OIL**	**14**	162.0557	6.01 ± 0.28	134.0597, 116.0491, 106.0648, 79.0541, 89.0383	20	+	+
	**15**	225.1488	6.44 ± 0.16	69.0698, 111.0797, 165.1268, 43.0183, 123.1160	20	+	+
	**16**	288.1237	10.05 ± 0.13	212.0713, 230.0797, 156.0798, 81.0701, 272.0908	35	+	+
	**17**	467.3510	20.42 ± 0.3	57.0699, 155.0107, 238.9294, 314.0962, 419.3015	45	+	+
	**18**	483.3824	22.01 ± 0.18	45.03042, 89.0596, 153.0190, 264.9438, 321.2125	45	-	+
	**19**	379.3365	24.26 ± 0.34	69.0698, 83.0854, 57.0701, 295.2418, 323.2717	20	-	+
	**20**	413.3429	24.81 ± 0.34	69.0708, 395.3291, 83.0859, 271.2046, 315.2671	30	+	+
	**21**	773.5184	27.95 ± 0.7	493.2745, 521.3057, 309.2407, 91.0394, 73. 0289	45	+	+
**SESAME OIL**	**22**	373.1286	8.71 ± 0.15	355.1166, 151.0383, 205.0850, 337.1064, 137.0588	10	-	+
	**23**	371.1136	10.6 ± 0.19	353.1019, 203.0700, 335.0913, 149.0231, 135.0440	10	+	+
	**24**	299.2592	12.88 ± 0.07	281.2471, 263.2366, 121.1008, 71.0491, 135.1165	10	-	+
	**25**	337.1082	13.66 ± 0.22	135.0438, 289.0856, 261.0903, 185.0590, 319.0959	20	+	+
	**26**	173.0604	14.53 ± 0.23	143.0485, 115.0539, 131.0487, 155.0483, 65.0381	10	+	+
**FLAX OIL**	**27**	212.0823	8.93 ± 0.26	167.0600, 195.0551, 140.0488, 185.0704, 97.9609	20	-	+
	**28**	287.2016	10.45 ± 0.08	121.0642, 93.0694, 105.0694, 148.0878, 79.0541	40	-	+
	**29**	285.1853	11.13 ± 0.1	148.0878, 187.1114, 105.0694, 133.0642, 199.1109	40	-	+

The highest intensities in all sunflower seed oil samples were observed for the markers of *m/z* 203.1076, *m/z* 392.2444, *m/z* 432.2758 and 434.2913. All the specific markers were detected in samples prepared with 1% sunflower oil (Table 1). In rapeseed oil samples, we detected eight specific markers, with the highest intensities observed for ions at *m/z* 413.3429, *m/z* 467.3510 and *m/z* 483.3824. Six of the eight markers were visible in samples containing 1% added rapeseed oil. In sesame seed oil, five specific metabolomic markers were detected, with the highest intensities ions at *m/z* 337.1082 and *m/z* 371.1136. Four of the six ions were observed in samples prepared with 1% sesame seed oil. In flaxseeds oil, the most intense ion was at *m/z* 212.0823. Yet it was not detected at 1%. All of the metabolomic markers charactersitic for each oil plant species were detected in mixed samples containing 5% of the analysed oil (Table 1).

The use of mass spectrometry for the detection of impurities in edible oil and confirmation of the authenticity of plant oils has been described by Catharino et al. [29], who determined, among other properties, the quality and adulteration of oils by using direct infusion electrospray ionization–mass spectrometry. They investigated different plant seed oils, including sunflower seed oil, and used fatty acid and phenolic compounds as factors for differentiating among individual oils [29]. Jergovic et al. [30] focused on investigating the adulteration of extra virgin olive oil by sunflower oil by using MALDI-TOF/MS fingerprinting of triacylglycerol profiles. Zhang et al. [31] used GC–MS analyses in SIM mode to study the classification of fatty acids in five oils, including sunflower, sesame and rapeseed oils, they were able to detected adulteration of oil with other oils at the level of 10%. Kotecka-Majchrzak et al. [32] described 92 specific peptide markers for 10 cold-pressed oils, including 21 for sesame, 18 for sunflower, 11 for flax and 1 for rapeseed oil, identified by UHPLC-Q-TOF-MS/MS analyses. All of these methods are based on finding specific compounds, characteristic of the oil type, allowing each oil to be distinguished from other oils and to detect adulteration under laboratory conditions.

### Abundance variability of markers

The analysis of marker abundancies showed that the ion intensity of specific metabolomic markers varies not only among oil samples from different producers, but also among different batches of the same product from the same company. To visualise this variability, the superimposed extracted ion chromatogram (EIC) of selected markers for eight samples (four manufacturers, two batch for each manufacturer) are presented in Fig 3 together with the fragmentation spectra of the markers. The differences in intensities were significant, e.g. for the ion of *m/z* 392.2444 from sunflower oil the difference between the samples with the highest and lowest marker MS signal was 89.2%, for the ion of *m/z* 413.3429 from rapeseed oil the difference was 47.1%, for the ion of *m/z* 299.2592 in sesame oil it was 88.1%, and for the ion of *m/z* 287.2016 specific for flaxeed oil it was 74.2%. These differences may be attributed to different regions of cultivation, weather conditions, water availability and the oil pressing method used.

To summarise briefly, our findings indicate that the metabolic markers will be successful in qualitative analyses, even at low concentration levels, and they can be used for oil authenticity testing and detection of adulterations. Yet if the quantitation was to be performed based on metabolic markers the uncertainty of such determinations will be very high.

### Total phenolic content, total carotenoid content and antioxidant activity

Cold pressed oils contain many groups of phytochemicals. The bioactive components of edible plant seed oils determine the quality of the oil, and the concentration of bioactive compounds depends on the processing conditions, among other factors. High temperature can lead to the degradation and loss of activity of bioactive compounds. The effect of bioactive compounds on human health also depends on their bioavailability and the amount consumed [33]. Here, we determined the antioxidant activity and total phenolic and carotenoid contents of the cold pressed sunflower seed oils, sesame oils, rapeseed oils and flaxseed oils to demonstrate oil quality and highlight differences among the oil types and producers.

#### Total phenolic content

The total phenolic content of unrefined, cold pressed seed oils analysed using the Folin–Ciocalteu colorimetric method was expressed as milligram gallic acid equivalents per kg (see Table 2). The highest concentration was observed in sesame seed oil (84.03 ± 4.19 − 103.79 ± 3.67 mg GA/kg). Sunflower seed oil had the lowest content of polyphenols (n.d., − 12.60 ± 0.09 mg GA/kg). We did not detect phenols in one sample of sunflower oil. Rapeseed oil and flaxseed oil were found to have a similar phenolic content: 20.43 ± 0.56–61.10 ± 0.76 mg GA/kg and 18.10 ± 0.42–45.12 ± 2.05 mg GA/kg, respectively. In a study by Toorani et al. [34], the total phenolic content (TPC) was lower in sesame seed oil (51.0 ± 1.4 mg/kg). In flaxseed oil originating from New Zealand, 136.93 mg GA/100 g and 87–307.3 mg ferulic acid per 100 g were reported [35, 36]. Samples from Poland contained 1.14 mg caffeic acid per 100 g, and the concentrations in sunflower and rapeseed oil were 1.20 and 1.31 mg CAE/100 g (CAE, caffeic acid equivalent), respectively [37].

**Table 2:** Trolox equivalent antioxidant capacity (TEAC), total phenolic content (TPC) and total carotenoid content (TCC) of the oil samples.

	Sample name	TEAC [mg Trolox/kg oil]	TPC [mg of gallic acid/kg oil]	TCC [mg β-carotene/kg oil]
**SUNFLOWER OIL**	S_a	252.22 ± 3.65^bcd^	4.33 ± 0.13^hi^	285.85 ± 3.47^jk^
	S_b	249.78 ± 5.30^cde^	4.24 ± 0.07^hi^	314.43 ± 2.59 ^ijk^
	S_c	222.44 ± 0.66^de^	12.6 ± 0.09^gh^	247.97 ± 5.23 ^k^
	S_d	282.38 ± 12.28^bcd^	n.d*^i^	256.55 ± 5.86 ^k^
**SESAME OIL**	Ses_a	181.65 ± 2.30^fgh^	94.57 ± 4.44^b^	340.49 ± 6.46^ij^
	Ses_b	148.10 ± 6.06^h^	84.03 ± 4.19^c^	286.36 ± 7.44^jk^
	Ses_c	219.54 ± 7.64^def^	99.05 ± 3.35^ab^	363.29 ± 5.89^i^
	Ses_d	165.36 ± 5.41^gh^	103.79 ± 3.67^a^	259.29 ± 6.22^k^
**RAPESEED OIL**	R_a	240.56 ± 1.82^cde^	20.43 ± 0.57^g^	3196.71 ± 64.5^a^
	R_b	202.85 ± 4.12^efg^	61.11 ± 0.76^d^	1253.74 ± 17.82^e^
	R_c	291.16 ± 1.98^ab^	48.20 ± 2.26^e^	3038.1 ± 56.81^b^
	R_d	244.85 ± 11.56^cde^	30.1 ± 1.06^f^	1638.73 ± 17.69^c^
**FLAXSEED OIL**	F_a	297.22 ± 2.32^a^	31.29 ± 1.20^f^	1501.91 ± 12.02^d^
	F_b	284.64 ± 3.90^abc^	18.10 ± 0.42^g^	1148.35 ± 14.92^f^
	F_c	269.30 ± 11.19^bcd^	44.76 ± 1.40^e^	927.03 ± 11.54^h^
	F_d	245.67 ± 7.59^cde^	45.13 ± 2.05^e^	1034.51 ± 9.31^g^

The results are presented as mean ± standard deviation (n = 3; Tukey’s HSD test; homogenous groups, alpha = 0.05). The values sharing a common letter do not differ significantly at p < 0.05; *n.d.- not detected

#### Total carotenoid content

Carotenoids are yellow-to-red pigments found in different plant organs, e.g. flowers, fruits and leaves [38]. Table 2 shows that the highest total carotenoid content (TCC) was found in rapeseed (1253.74–3196.71 mg/kg) and flaxseed (927.03–1501.91 mg/kg) oils, and these oils showed the greatest differences in carotenoid contents between four different manufacturers. Analyses of rapeseed oil by Rafałowski et al. [39] showed 80.9 mg/kg TCC, whereas Tańska et al. [40] reported 52.6 mg/kg and Konuskan et al. [38] reported 12.01 mg/kg. A similar situation was observed for other oils, with unrefined, cold-pressed flaxseed oil having reported TCC values of 150.1 mg/kg [39] and 47.6 mg/kg [40] in laboratory studies. The lowest value in our research, 247.97 mg/kg, was observed for sunflower oil from one of the manufacturers and was higher than the 0.7 mg/kg recorded by Franke et al. [41] or the results of a study by Rafałowski et al. [39], who found no β-carotene.

#### Antioxidant activity

The bioactive compounds in plant seed oil influence the antioxidant properties of the oil. We expressed the ABTS radical scavenging activity in mg Trolox per kg and the results for the antioxidant activity of each neat oil are presented in Table 2. Application of ABTS assay enabled evaluation of the total antioxidant potential, taking into account the activity of both lipophilic and more hydrophilic compounds. The highest antioxidant activity was observed for flaxseed oil, with samples from different manufacturers showing the smallest differences among the four oil types tested, ranging from 245.67 ± 7.59 to 297.22 ± 2.32 mg Trolox/kg oil. Similar to flaxseed oil, a high antioxidant potential was observed for sunflower and rapeseed oils, ranging from 222.44 ± 0.66 to 282.38 ± 12.28 mg Trolox/kg oil and from 202.85 ± 4.12 to 291.16 ± 1.98 mg Trolox/kg oil, respectively. The lowest values were recorded for sesame oil, where the activity range, depending on the oil manufacturer, was 148.10 ± 6.06 – 219.54 ± 7.64 mg Trolox/kg oil. Kostadinović Velićkovska et al. [42] analysed oils obtained from seeds grown in Macedonia and observed that the highest antioxidant properties were in sesame (87.3 ± 0.2 Trolox mg/l oil) and flaxseed (64.7 ± 0.7 Trolox mg/l oil) oils, followed by sunflower oil (13.2 ± 4.1 Trolox mg/l oil). For comparison, the results of the DPPH (1,1-diphenyl-2-picrylhydrazyl) free-radical scavenging activity assay for flaxseed oil from Poland provided an antioxidant potential of 1.10–2.30 mM TEAC/kg [43]. Using the same method, Riberio et al. [44] analysed sesame oil obtained by pressing, and observed 88.85 ± 2.57 μmol Trolox/g of oil. Oil obtained from unroasted rapeseed with a 10% moisture content provided an antioxidant activity of 269.11 ± 2.23 μM Trolox/g [45].

Our results obtained using spectrometric analyses showed differences between plant species and manufacturers, and our results differed from those obtained by other researchers. These variations could be due to many factors, including the plant species and growth conditions such as climate, sun exposure, degree of maturity, harvesting technology, irrigation and the quality of the soils in which the oil seed crops were grown [13]. The quality of an oil also depends on the grain preparation techniques, the pressing process and the oil extraction technique. Significant differences in antioxidant activity, total phenolic and carotenoid content between oil types and between oils from different manufacturers were observed. Thus any adulterating of oils with other ones may also result in the change of content of bioactive components. In the light of our findings, adding sunflower oil to sesame or flaxseed oil will apparently reduce the total phenolic content, whereas adding sunflower or sesame oil to rapeseed or flaxseed will result in decreasing the total content of carotenoids. The potential reduction in oil quality resulting from changes in the content of phytochemicals may affect the health of consumers who, unaware of the adulteration of products, may experience negative consequences not only from the lowered bioactivity of a consumed oil but also from the presence of allergens they are allergic to. If oils were to use for pharmaceutical purposes or dietotherapy the products should be thoroughly tested, the detailed composition and bioactive properties should be determined and placed on the product label or included in the product characteristic card. Methods for oil composition and bioactivity control as well as methods for testing the authenticity and detecting adulterations of oils should be validated and standardised.

## Conclusions, limitations and perspectives

In this study metabolomic approach based on liquid chromatography coupled to quadrupole-time-of-flight mass spectrometry was employed to discover oil-specific metabolomic markers for four unrefined, cold-pressed seed oils. The set of 29 markers involving 13 markers for sunflower, 8 for rapeseed, 5 for sesame and 3 for flaxseed oil was determined. Markers were annotated to the level of retention times and characteristic ions, i.e. accurate masses of precursor and fragment ions. The structure of these compound are yet to be elucidated. All detected markers can be used as qualitative markers to confirm the authenticity or to detect the adulteration of plant oils. The reference materials, certified oils samples or samples of oils obtained from highly trusted producers, will be sufficient to be used for validation purposes and demonstrating the implemented method applicability and utility. To build the system of the effective control of edible oils, inter-laboratory proficiency testing programs focusing on new omics methodologies adopted to edible oil authenticity testing should also be developed.

## Supporting information

S1 File ZIP file containing i) an excel file of metabolic features of oils used for PCA and OPLS-DA analyses, ii) a Word file listing samples and CEF files, and iii) CEF files of LC-QTOF MS profiling of oils. (ZIP)
