# Supplementary material for: Identification of sunflower, rapeseed, flaxseed and sesame seed oil metabolomic markers as a potential tool for oil authentication and detecting adulterations
Source: PLoS One. 2023 Apr 20;18(4):e0284599. doi: 10.1371/journal.pone.0284599 (PMC10118080; doi:10.1371/journal.pone.0284599)
Supplement: S1 File — (ZIP) [file pone.0284599.s001.zip › Data_information.docx]

**CEF**

**Sunflower oil**

S_a1

S_a2

S_b1

S_b2

S_c1

S_c2

S_d1

S_d2

**Rapeseed oil**

R_a1

R_a2

R_b1

R_b2

R_c1

R_c2

R_d1

R_d2

**Flaxseed oil**

F_a1

F_a2

F_b1

F_b2

F_c1

F_c2

F_d1

F_d2

**Sesame seed oil**

Ses_a1

Ses_a2

Ses_b1

Ses_b2

Ses_c1

Ses_c2

Ses_d1

Ses_d2
